# Role of NKp46^+^ natural killer cells in house dust mite‐driven asthma

**DOI:** 10.15252/emmm.201708657

**Published:** 2018-02-15

**Authors:** Eline Haspeslagh, Mary J van Helden, Kim Deswarte, Sofie De Prijck, Justine van Moorleghem, Louis Boon, Hamida Hammad, Eric Vivier, Bart N Lambrecht

**Affiliations:** ^1^ Immunoregulation and Mucosal Immunology VIB Center for Inflammation Research Ghent Belgium; ^2^ Department of Biomedical Molecular Biology Ghent University Ghent Belgium; ^3^ Department of Internal Medicine Ghent University Ghent Belgium; ^4^ Bioceros BV Utrecht The Netherlands; ^5^ Centre d'Immunologie de Marseille‐Luminy Inserm, CNRS Aix Marseille Université Parc Scientifique & Technologique de Luminy Marseille Cedex France; ^6^ Service d'Immunologie Hôpital de la Timone Assistance Publique‐Hôpitaux de Marseille Marseille France; ^7^ Department of Pulmonary Medicine Erasmus MC Rotterdam The Netherlands; ^8^ Present address: Aduro Biotech Europe Oss The Netherlands

**Keywords:** allergic asthma, house dust mite, NK cells, NKG2D, NKp46, Immunology, Respiratory System

## Abstract

House dust mite (HDM)‐allergic asthma is driven by T helper 2 (Th2) lymphocytes, but also innate immune cells control key aspects of the disease. The precise function of innate natural killer (NK) cells during the initiation and propagation of asthma has been very confusing, in part because different, not entirely specific, strategies were used to target these cells. We show that HDM inhalation rapidly led to the accumulation of NK cells in the lung‐draining lymph nodes and of activated CD69^+^ NK cells in the bronchoalveolar lumen. However, genetically engineered *Ncr1*‐DTA or *Ncr1*‐DTR mice that constitutively or temporarily lack NK cells, still developed all key features of acute or chronic HDM‐driven asthma, such as bronchial hyperreactivity, Th2 cytokine production, eosinophilia, mucus overproduction, and Th2‐dependent immunoglobulin serum titers. The same results were obtained by administration of conventional NK1.1 or asialo‐GM1 NK cell‐depleting antibodies, antibody‐mediated blocking of the NKG2D receptor, or genetic NKG2D deficiency. Thus, although NK cells accumulate in allergen‐challenged lungs, our findings comprehensively demonstrate that these cells are not required for HDM‐driven asthma in the mouse.

## Introduction

Asthma is a major and ever‐increasing health problem that currently affects 300 million people worldwide (Lambrecht & Hammad, 2017). Allergic asthma is a prototype of type 2 immunity, orchestrated by an aberrant adaptive CD4^+^ T helper 2 (Th2) cell immune response to airborne allergens such as house dust mite (HDM). Th2 cells produce the cytokines IL‐4, IL‐5, IL‐9, and IL‐13, which induce immunoglobulin E (IgE) production by B cells, eosinophil infiltration of the airways, basophil and mast cell activation, and goblet cell hyperplasia and increased mucus production (Lambrecht & Hammad, 2014). The excessive inflammatory response causes bronchial hyperreactivity (BHR) and creates breathing difficulties. Although Th2 cells have historically been regarded as central mediators, several innate immune cells are also critically important in orchestrating various aspects of the allergic inflammatory response in mouse models (Akbari *et al*, 2003; Sokol *et al*, 2008; Hammad *et al*, 2009, 2010; Nussbaum *et al*, 2013; Plantinga *et al*, 2013; Halim *et al*, 2014; Schuijs *et al*, 2015).

Conventional natural killer (NK) cells are key components of innate immunity, best known for their anti‐viral and anti‐tumor activity. Moreover, NK cells are increasingly appreciated to play a regulatory role in the immune system, being capable of influencing DC functions (Degli‐Esposti & Smyth, 2005; Walzer *et al*, 2005; Moretta *et al*, 2006), shaping CD8^+^ T cell memory responses (Soderquest *et al*, 2011; Crouse *et al*, 2014; Xu *et al*, 2014), and promoting CD4^+^ Th1 cell polarization (Martín‐Fontecha *et al*, 2004; Morandi *et al*, 2006; Lu *et al*, 2007). Under certain conditions, NK cells can even secrete Th2 cytokines, and thereby might promote or enhance type 2 immunity (Warren *et al*, 1995; Walker *et al*, 1998; Cooper *et al*, 2011).

NK cells are ubiquitously present in human and mouse lungs, where they comprise 10–15% of resident lymphocytes (Gregoire *et al*, 2007; Marquardt *et al*, 2017). Therefore, many studies have aimed at elucidating their role in allergic asthma. Initial studies have used NK1.1 or asialo‐GM1 (ASGM1) depleting antibodies (Korsgren *et al*, 1999; Ple *et al*, 2010), or mice genetically deficient for the activating receptor NKp46 (Ghadially *et al*, 2013) or NKG2D (Farhadi *et al*, 2014). These studies showed that NK cells were crucial and non‐redundant for asthma development induced by ovalbumin (OVA)/alum (Korsgren *et al*, 1999; Ple *et al*, 2010; Ghadially *et al*, 2013) or chronic HDM exposure (Farhadi *et al*, 2014). In contrast, increased pulmonary NK cell numbers were recently correlated with reduced HDM‐mediated airway inflammation in mice (Ferrini *et al*, 2016; Simons *et al*, 2017). Moreover, antibody‐mediated NK cell depletion was shown to increase eosinophilia and goblet cell hyperplasia in early papain‐ or bleomycin‐induced lung inflammation (Wan Jiacheng Bi *et al*, 2017). Overall, results on the role of NK cells in experimental asthma are thus conflicting, model‐dependent, and, most importantly, have not used the same tools for NK cell depletion or alteration of function.

To definitively address the role of NK cells in HDM‐driven allergic asthma, we here performed a comprehensive study using various approaches to target or deplete NK cells in a well‐established HDM asthma model. In addition to conventional antibody‐mediated depletion techniques, we also included novel tools to genetically deplete NKp46^+^ NK cells (Narni‐Mancinelli *et al*, 2011). Although NK cells were activated in the bronchoalveolar lumen (BAL) of mice exposed to HDM, constitutive or temporary genetic depletion of NK cells did not reduce or enhance HDM‐induced asthma features. This was confirmed in a more chronic asthma model and using another allergen. In our hands, also NKG2D‐deficient mice developed normal HDM‐induced eosinophilia. Taken together, our findings challenge earlier findings and show that NK cells play a minor role in the development of HDM‐induced allergic asthma.

## Results

### HDM allergen activates NK cells in selected compartments

**Figure 1:**
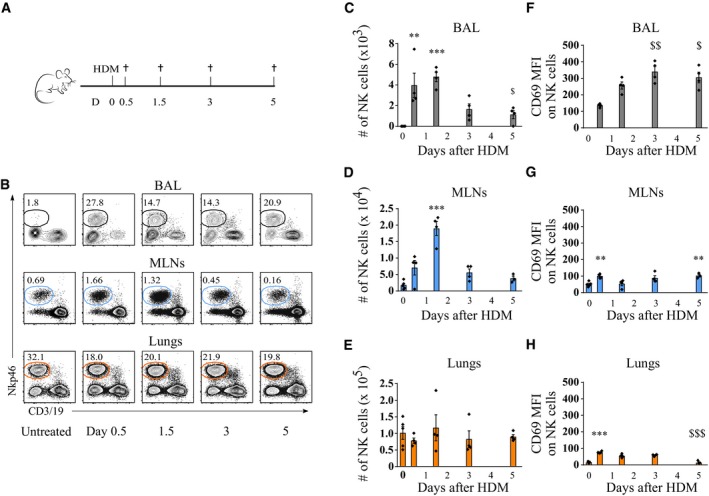
Pulmonary HDM exposure leads to infiltration of NK cells in bronchoalveolar lavage fluid (BAL) and mediastinal lymph nodes (MLNs) AEight‐week‐old female C57Bl/6J mice were administered 100 μg HDM intratracheally. BAL, MLNs, and lung tissue were harvested on indicated time points for analysis by flow cytometry.BNK cell infiltration in BAL, MLN single‐cell suspensions, and homogenized lung tissue, pre‐gated on live CD45^+^ single cells. One representative plot is shown for each condition, and percentage of NK cells (live, CD45^+^CD3/19‐Nkp46^+^) of live CD45^+^ cells is indicated.C–EQuantification of NK cell infiltration in BAL (C), MLN cell suspensions (D), and homogenized lung tissue (E).F–HMean fluorescence intensity (MFI) of activation marker CD69 on NK cells.Data information: Data were analyzed with an unpaired Kruskal–Wallis test without multiple comparison correction, and individual data points are shown ± SEM.. *N* = 5 mice for D0 and 4 for other time points. Results are representative of at least two independently performed experiments. **P < 0.01; ***P < 0.001 compared to day 0. ^$^ *P* < 0.05; ^$$^ *P* < 0.01; ^$$$^ *P* < 0.001 compared to day 0.5. All exact *P*‐values are presented in Table EV1.

We first analyzed the impact of HDM exposure on NK cell numbers and activation in the lung interstitium, bronchoalveolar lumen accessible by lavage (BAL), and lung‐draining mediastinal lymph nodes (MLNs). C57Bl/6 mice received an intratracheal (i.t.) instillation of a high dose of crude HDM extract, known to induce an innate immune response (Hammad *et al*, 2010), and NK cell responses were assessed over time (Fig 1A). In naïve mice, very few NK cells were located in BAL and MLNs (Fig 1B–D). From day 0.5 after HDM exposure, NK cells were easily detectable in both organs and numbers peaked at day 1.5, followed by a rapid decline. The expression of the activation marker CD69 on BAL, but not MLN, NK cells increased over time, suggesting an enhanced activation state (Fig 1F and G). In lung tissue from which circulating blood cells were flushed out, NK cell numbers were substantial in naïve mice (Fig 1B and E), consistent with earlier reports (Gregoire *et al*, 2007), and were not significantly influenced by HDM instillation. Lung NK cells did, however, temporarily gain some CD69 expression (Fig 1H). Thus, HDM instillation resulted in a temporary infiltration and activation of NK cells in various anatomical regions of the pulmonary immune system, including the inductive MLN site.

### Absence of NKp46^+^ cells does not affect allergic asthma induced by several models

**Figure 2:**
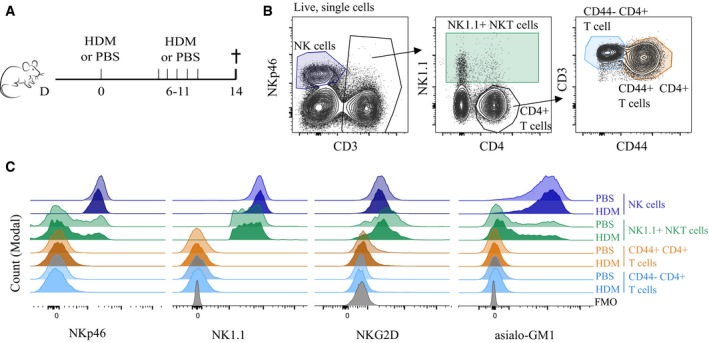
NK cell marker expression on pulmonary T, NKT, and NK cells remains unchanged in response to HDM‐mediated allergic asthma C57Bl/6J mice were sensitized intratracheally on day 0 with 1 μg HDM or PBS, followed by five consecutive intranasal challenges with 10 μg HDM or PBS 7 days later. Lungs were harvested and homogenized 4 days after the last challenge.Gating strategy for identification of NK cells, NK1.1^+^ NKT cells, and naïve (CD44^−^) and memory (CD44^+^) CD4^+^ T cells.Representative histogram of NKp46, NK1.1, NKG2D, and asialo‐GM1 expression on cell populations gated as in (B). Data are representative of two experiments with each two to three mice per group. FMO = Fluorescence minus one.

Several studies have been performed to address the role of NK cells in allergic asthma, using various markers to target or deplete NK cells (Korsgren *et al*, 1999; Ple *et al*, 2010; Ghadially *et al*, 2013; Farhadi *et al*, 2014). Some of these markers are also expressed by subsets of T cells in various inflammatory conditions (Slifka *et al*, 2000). Since activated CD4^+^ T cells are key players in allergic asthma, we determined the specificity of expression of NKp46, NKG2D, ASGM1, and NK1.1 on NK cells, NK1.1^+^ natural killer T (NKT) cells, and naïve and memory conventional CD4^+^ T cells in the lungs of naïve and asthmatic mice (Fig 2A and B). NKp46 was expressed on all NK cells, and a minor subset of NK1.1^+^ NKT cells, as previously reported (Walzer *et al*, 2007; Yu *et al*, 2011), whereas expression on naïve or memory T cells was absent in both groups of mice (Fig 2C). NK1.1, NKG2D, and ASGM1 were all highly expressed on NK cells, and particularly NK1.1 and NKG2D were also highly expressed on most NK1.1^+^ NKT cells. Thus, to deplete NK cells, NKp46 was first chosen based on its robust expression on all NK cells and limited expression on other cell types relevant to asthma.

**Figure EV1:**
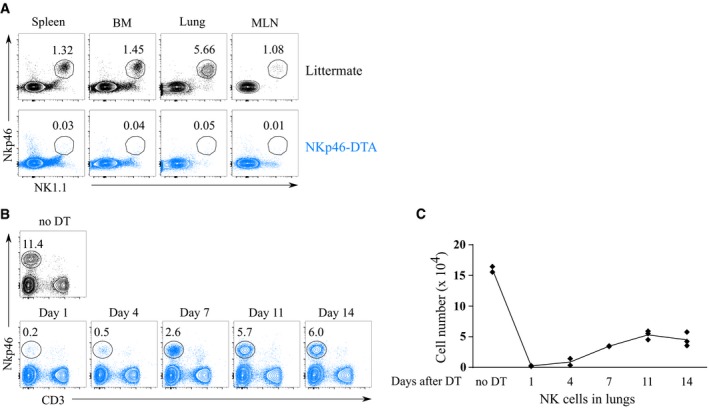
NK cells are efficiently depleted in *Ncr1* ^iCre/+^ *ROSA*^DTA^ ^/+^ mice and can efficiently be depleted by DT injection in *Ncr1* ^iCre/+^ *ROSA*^DTR^ ^/+^ mice AFlow cytometry analysis of NK cells in spleen, bone marrow (BM), lung, and mediastinal lymph nodes (MLN) isolated from naïve NKp46‐DTA (*Ncr1* ^iCre/+^ *ROSA* ^DTA/+^) mice or littermate controls (*Ncr1* ^iCre/+^ *ROSA* ^+/+^). First panel was pre‐gated on live CD45^+^CD3^−^ single cells.B, CNKp46‐DTR (*Ncr1* ^iCre/+^ *ROSA* ^DTR/+^) mice were treated with 200 ng DT intravenously and sacrificed at indicated time points. (B) Representative flow cytometry plots and (C) quantification of NK cell numbers (live, CD3^−^ NKp46^+^) in lung tissue cell suspensions. Gated on live single cells. *N* = two to three mice per time point and means are connected.

**Figure 3:**
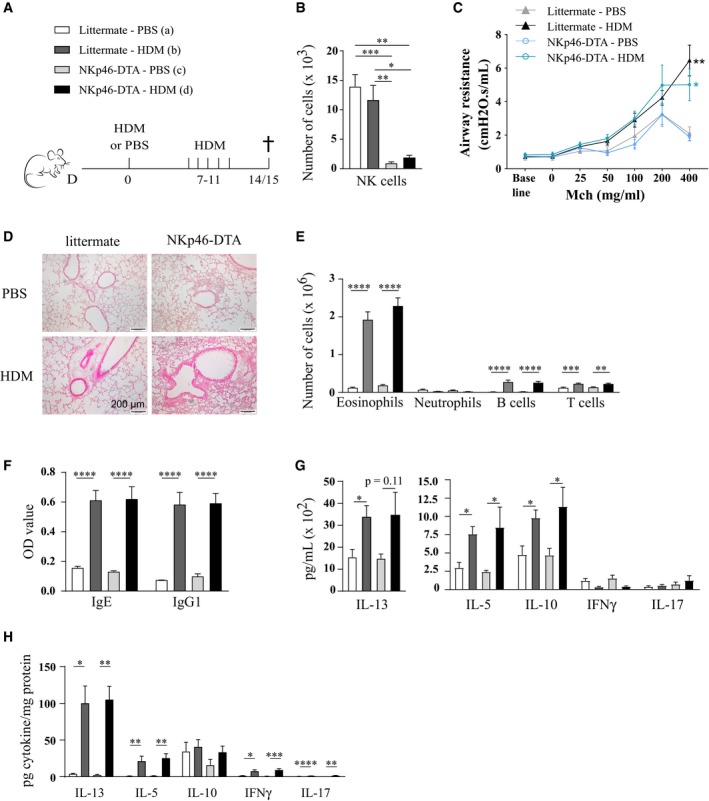
Absence of NKp46^+^ cells does not reduce nor exacerbate hallmarks of HDM‐induced allergic asthma On indicated time points, mice deficient of NKp46^+^ cells (NKp46‐DTA, *Ncr1* ^iCre/+^ *ROSA* ^DTA/+^) and littermate controls (*Ncr1* ^iCre/+^ *ROSA* ^+/+^) were sensitized intratracheally with 1 μg HDM, or mock‐sensitized with PBS, followed by five consecutive intranasal challenges with 10 μg HDM. 3–4 days later, bronchial hyperreactivity (BHR) was measured (C) or organs were harvested for analysis (B, D–G). Alternatively, lung tissue was harvested 3 h after the 4th challenge on day 10 (H).NK cell (live, TCR‐β^−^ NK1.1^+^ CD122^+^) numbers in BAL, assessed by flow cytometry. *n* = 7 (a, b) or 6 (c, d).Airway resistance in response to increasing doses of methacholine (Mch). Data are pooled from two independent experiments, with total *n* = 7 (a), 14 (b), 7 (c) and 15 (d). *P = 0.0348; **P = 0.0058 for dose 400 mg/ml Mch compared to mock‐sensitized control group (d to c and b to a, respectively).Detection of mucus production by periodic acid–Schiff (PAS) staining on OCT‐inflated lung cryosections.Infiltration of eosinophils, neutrophils, B cells, and T cells to BAL, assessed by flow cytometry. Data are pooled from four independent experiments with total *n* = 17 (a), 28 (b), 19 (c), and 26 (d).HDM‐specific immunoglobulin serum levels, detected by ELISA. Data are pooled from three independent experiments with total *n* = 13 (a), 21 (b), 17 (c), and 20 (d).MLN single‐cell suspensions were restimulated with 15 μg/ml HDM for 3 days, and cytokine production was measured by ELISA. Data are representative of four independent experiments. *n* = 7 (a, b) or 6 (c, d).Snap‐frozen lung tissue was homogenized and analyzed for cytokine levels by ELISA, and for total protein content by NanoOrange technology. Results are depicted as pg cytokine/mg total protein. *n* = 5 (a, c) or 6 (b, d).Data information: All data were analyzed with an unpaired Kruskal–Wallis test without multiple comparison correction and are shown as means ± SEM. *P < 0.05; **P < 0.01; ***P < 0.001; ****P < 0.0001. Exact *P*‐values are presented in Table EV1.

ROSA‐flox‐stop‐flox‐diphtheria toxin A (*ROSA* ^DTA/DTA^) mice were crossed to mice that express Cre recombinase driven by the NKp46 promoter (*Ncr1* ^iCre/iCre^) (Narni‐Mancinelli *et al*, 2011; Deauvieau *et al*, 2016). *ROSA* ^DTA/+^ *Ncr1* ^*i*Cre/+^ mice, called NKp46‐DTA hereafter, were confirmed to lack NKp46^+^ NK1.1^+^ CD3^−^ cells in all investigated organs (Fig EV1A). These cells include bone fide NK cells, but also subsets of innate lymphoid cells (ILCs) type 1 (ILC1s) and ILC3s (Cella *et al*, 2009; Luci *et al*, 2009; Sanos *et al*, 2009; Cortez & Colonna, 2016). NKp46‐DTA mice, and their littermate controls (*ROSA* ^+/+^ *Ncr1* ^iCre/+^), were i.t. sensitized to HDM and subsequently challenged with intranasal (i.n.) HDM inoculations on five repetitive days (Fig 3A). NKp46‐DTA mice lacked NK cells in BAL (Fig 3B). HDM‐sensitized, but not mock‐sensitized, littermate mice exhibited strong bronchial hyperreactivity (BHR) in response to increasing doses of methacholine, and this response was also seen in NKp46‐DTA mice (Fig 3C). In both littermate control and NKp46‐DTA mice, HDM sensitization induced peribronchial and perivascular influx of immune cells in the lungs (Fig 3D), increased mucus production (Fig 3D), and eosinophil, B cell, and T cell infiltration in the BAL (Fig 3E), hallmarks of type 2 immunity. Moreover, serum levels of Th2‐associated HDM‐specific immunoglobulins E (IgE) and IgG1 were not affected by NKp46^+^ cell absence (Fig 3F). The induction of eosinophilia and goblet cell metaplasia depends on IL‐5 and IL‐13, respectively. We therefore measured the presence of these key Th2 cytokines in total lung tissue, and their production by HDM‐restimulated MLN cells. The levels of these and other cytokines (IL‐10, IL‐17, IFN‐γ) were comparable between NKp46‐DTA and littermate mice (Fig 3G and H).

**Figure 4:**
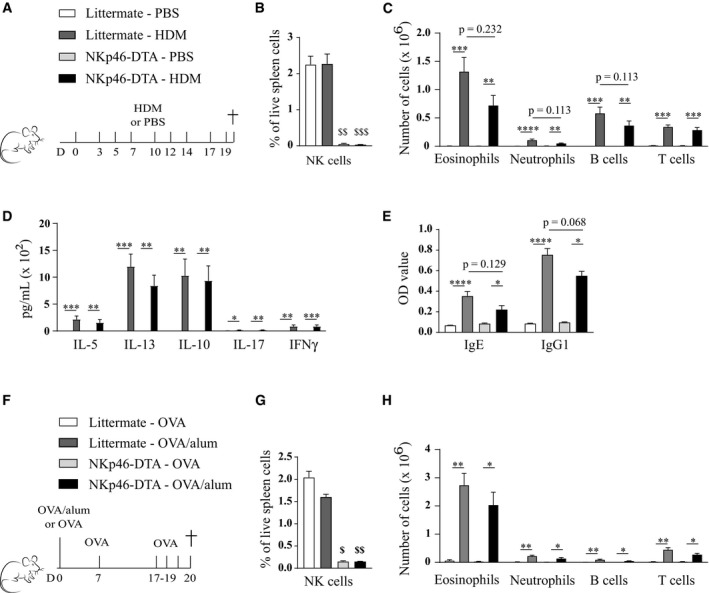
Absence of NKp46^+^ cells does not lead to a significant reduction in asthma hallmarks in response to chronic HDM exposure or OVA/alum‐induced allergic asthma Mice deficient of NKp46^+^ cells (NKp46‐DTA, *Ncr1* ^iCre/+^ *ROSA* ^DTA/+^) and littermates (*Ncr1* ^iCre/+^ *ROSA* ^+/+^) were intranasally challenged with 25 μg HDM three times a week for 3 weeks, or mock‐challenged with PBS, and analyzed 24 h after the last challenge (B–E).NK cell (live, TCR‐β^−^ NK1.1^+^ CD122^+^) absence was confirmed by flow cytometry on spleen cells.Infiltration of eosinophils, neutrophils, B cells, and T cells in BAL, assessed by flow cytometry.MLN single‐cell suspensions were restimulated with 15 μg/ml HDM for 3 days, and cytokine production was measured by ELISA.HDM‐specific immunoglobulin serum levels, detected by ELISA.On indicated time points, NKp46‐DTA mice and littermates intraperitoneally (i.p.) received 10 μg OVA absorbed on 1 mg alum (or OVA without alum as control), an i.p. boost of 10 μg OVA, and three intranasal OVA challenges (20 μg). Parameters were analyzed 24 h after the last challenge (G, H).NK cell (live, TCR‐β^−^ NK1.1^+^ CD122^+^) absence was confirmed by flow cytometry on spleen cells.Infiltration of eosinophils, neutrophils, B cells, and T cells in BAL, assessed by flow cytometry.Data information: Data in (B–E) are pooled from two independent experiments with total *n* = 6 for mock‐sensitized groups and total *n* = 14 for HDM‐sensitized groups. Data in (G, H): *n* = 4 for mock‐sensitized and *n* = 7 for OVA/alum‐sensitized groups. Data were analyzed with an unpaired Kruskal–Wallis test without multiple comparison correction and are shown as means ± SEM. *P < 0.05; **P < 0.01; ***P < 0.001; ****P < 0.0001. ^$^ *P* < 0.05; ^$$^ *P* < 0.01; ^$$$^ *P* < 0.001 compared to both littermate groups. Exact *P*‐values are presented in Table EV1.

Additionally, the response of NKp46‐DTA mice was assessed in a more chronic HDM‐driven model, adapted from Johnson *et al* (2004) and Gregory *et al* (2009), in which the mice were administered 25 μg HDM i.n. three times a week, for 3 weeks (Fig 4A and B). Again, the absence of NKp46^+^ cells did not significantly influence cardinal features of HDM‐driven asthma (Fig 4C–E), although there was a trend toward lower HDM‐specific IgG1 serum levels in NKp46‐DTA mice. These findings contrast with earlier studies in OVA/alum‐based allergic asthma models (Korsgren *et al*, 1999; Ple *et al*, 2010; Ghadially *et al*, 2013). To exclude allergen‐dependency, littermate and NKp46‐DTA mice were subjected to an OVA/alum‐based allergic asthma model (Fig 4F and G). Again, we found that inflammatory cell infiltration in BAL upon OVA/alum‐induced asthma was comparable between littermate and NKp46‐DTA mice (Fig 4H). Taken together, NKp46‐DTA and littermate control mice displayed a very similar Th2‐mediated asthmatic response to HDM or OVA.

### Cardinal features of HDM‐induced allergic asthma are not influenced by NKp46^+^ cell absence during the sensitization or challenge phase only

In NKp46‐DTA mice, ablation of NKp46^+^ NK cells occurs early during their maturation in the bone marrow (Narni‐Mancinelli *et al*, 2011), raising the possibility of functional replacement of NK cells by other immune cell types during immune development. Moreover, previous studies have suggested that NK cells can fulfill opposing roles during asthma, depending on the models used (Korsgren *et al*, 1999; Ple *et al*, 2010; Ghadially *et al*, 2013; Farhadi *et al*, 2014; Ferrini *et al*, 2016; Simons *et al*, 2017; Wan Jiacheng Bi *et al*, 2017). We therefore considered the possibility that NK cells might have contrasting roles in the sensitization and challenge phase of the HDM‐induced asthma model, which might be obscured in animals constitutively lacking NK cells. The influence of these two hypotheses was investigated by crossing *Ncr1* ^iCre/iCre^ mice to ROSA‐stop‐flox‐stop‐diphtheria toxin receptor (*ROSA* ^DTR/DTR^) mice (Narni‐Mancinelli *et al*, 2011), generating a model of inducible and temporary NK cell depletion. DT injection efficiently depleted pulmonary NK cells in *ROSA* ^DTR/+^ *Ncr1* ^iCre/+^ mice, called NKp46‐DTR hereafter, but not in DTR‐negative (*ROSA* ^+/+^ *Ncr1* ^iCre/+^) littermate mice, and the cells remained absent for at least 4 days (Fig EV1B and C).

**Figure 5:**
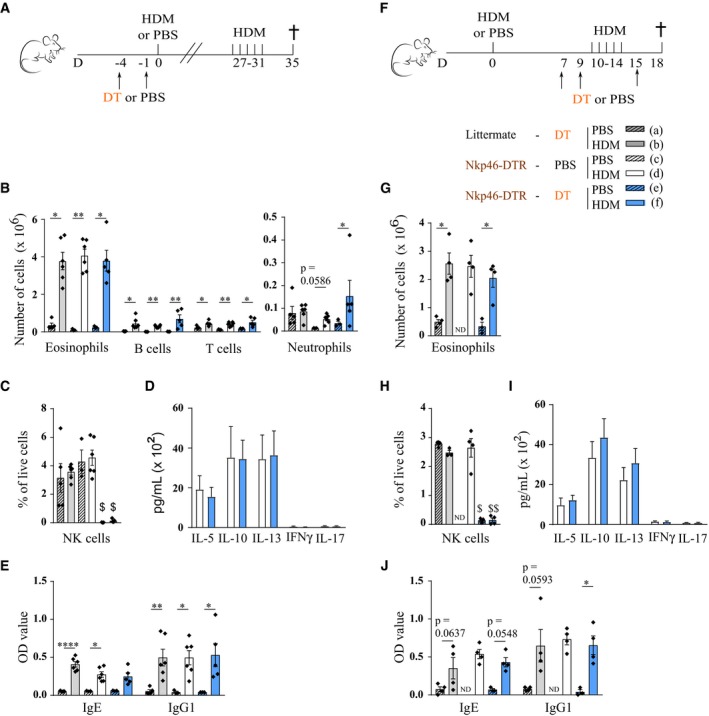
Induced absence of NKp46^+^ cells during the sensitization or challenge phase of HDM‐mediated allergic asthma does not influence asthma hallmarks A‐J(A, F) C57Bl/6 NKp46‐DTR mice (*Ncr1* ^iCre/+^ *ROSA* ^DTR/+^) or littermate controls (*Ncr1* ^iCre/+^ *ROSA* ^+/+^) were sensitized intratracheally on day 0 with 1 μg HDM, or mock‐sensitized with PBS. Four weeks (A) or 10 days (F) later, they were intranasally challenged on five consecutive days with 10 μg HDM. DT was injected at indicated time points, to deplete NKp46^+^ cells during the sensitization (A) or challenge (F) phase. Hallmarks of asthma were studied 3–4 days after the last challenge and shown in (B–E) and (G–J), respectively. (C, H) NK cell (live, TCR‐β^−^ NK1.1^+^ CD122^+^) depletion was confirmed by flow cytometry on spleen cells. (B, G) Infiltration of eosinophils, neutrophils, B cells, and T cells in BAL, determined by flow cytometry. (D, I) MLN single‐cell suspensions were restimulated with 15 μg/ml HDM for 3 days, and cytokine production was measured by ELISA. (E, J) HDM‐specific immunoglobulin serum levels, detected by ELISA.Data information: In (B, C, and E), *n* = 3 (c, e), 5 (f) or 6 (a, b, d); in (G, H, and J), *n* = 3 (e) or 4 (a, b, d, f); in (D and I), *n* = 5. BAL data are representative of two independently performed experiments. Data were analyzed with an unpaired Kruskal–Wallis test without multiple comparison correction, or with an unpaired Mann–Whitney test for (D and I), and are shown as means ± SEM. *P < 0.05; **P < 0.01; ****P < 0.0001; ^$^ *P* < 0.05; ^$$^ *P* < 0.01 for ≥ 2 control groups. All exact *P*‐values are presented in Table EV1.

**Figure EV2:**
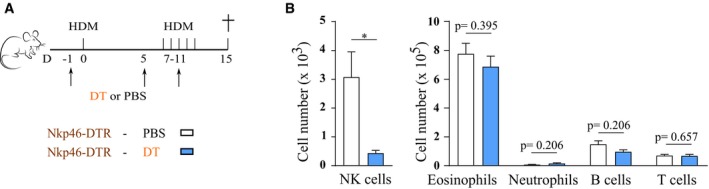
Ablation of NKp46^+^ cells during the entire HDM‐induced allergic asthma protocol does not influence immune cell infiltration to the BAL NKp46‐DTR mice (*Ncr1* ^iCre/+^ *ROSA* ^DTR/+^) were sensitized intratracheally on day 0 with 1 μg HDM, and 1 week later, they were intranasally challenged on five consecutive days with 10 μg HDM. DT was injected at indicated time points to deplete NKp46^+^ cells.Infiltration of NK cells, eosinophils, neutrophils, B cells, and T cells to BAL, determined by flow cytometry. Data are pooled from two independently performed experiments with total *n* = 11 (PBS group) or 8 (DT group). Data were analyzed with an unpaired Mann‐Whitney U‐test and are shown as means ± SEM. *P = 0.0157.

To investigate the role of NKp46^+^ cells during the allergic sensitization phase, NKp46‐DTR mice and littermate controls were treated with DT before HDM sensitization, or NKp46‐DTR mice were mock‐treated with PBS, as an additional control (Fig 5A). NK cell depletion was confirmed non‐invasively on blood samples (Fig 5C). NK cells were then allowed to reconstitute, and the mice were challenged with HDM 1 month after sensitization. All three groups of HDM‐sensitized and HDM‐challenged mice showed similar eosinophil, B cell, T cell, and neutrophil infiltration in the BAL (Fig 5B), and HDM‐specific IgE and IgG1 levels in serum (Fig 5E). Similarly, MLN cells harvested from DT‐treated and control PBS‐treated NKp46‐DTR mice produced comparable amounts of Th2 cytokines after HDM restimulation (Fig 5D). Next, NKp46^+^ cell function during the challenge phase was studied by injecting DT just before and during the HDM challenges (Fig 5F). Although this resulted in efficient NK cell depletion (Fig 5H), it did neither abort nor exacerbate BAL eosinophil infiltration (Fig 5G), cytokine production by HDM‐restimulated MLN cells (Fig 5I), or HDM‐specific IgE and IgG1 production (Fig 5J). Finally, NKp46‐DTR mice were treated with DT or PBS both before HDM sensitization and before HDM challenges, and here again, BAL immune cell infiltration was similar in the two groups of HDM‐sensitized and ‐challenged mice (Fig EV2A and B).

### Targeting NK cells with other (depletion) techniques does not affect HDM‐induced allergic asthma

**Figure 6:**
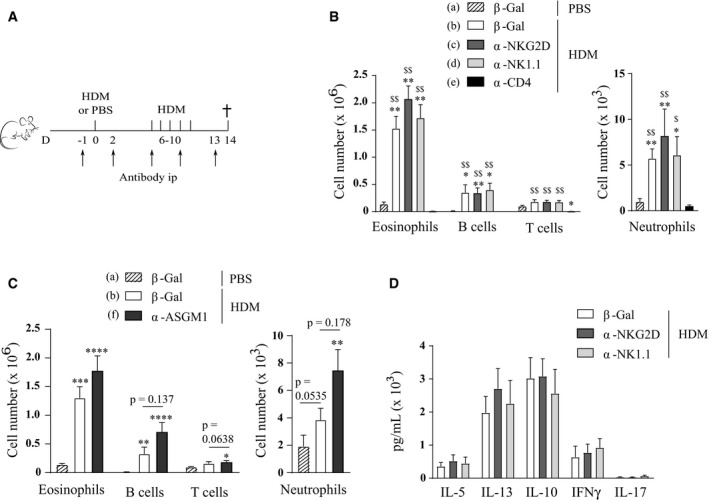
HDM‐induced allergic asthma is not influenced by antibody‐mediated depletion of NK cells or NKG2D blocking on NK cells AC57Bl/6J WT mice were sensitized intratracheally on day 0 with 1 μg HDM, or mock‐sensitized with PBS, followed after 1 week by five consecutive intranasal challenges with 10 μg HDM. From day −1, NK cell‐depleting anti‐NK1.1 or anti‐ASGM1, NKG2D‐blocking anti‐NKG2D, CD4^+^ T cell‐depleting anti‐CD4, or control anti‐β‐galactosidase antibodies were administered intraperitoneally every 3–4 days. Mice were analyzed 4 days after the last HDM challenge.B, CInfiltration of eosinophils, B cells, T cells, and neutrophils in BAL, assessed by flow cytometry. Data are pooled from two independently performed experiments with total *n* = 6 (a, e), 10 (c), 11 (b) or 12 (d) in (B) and total *n* = 11 (a), 12 (f) or 13 (b) in (C).DMLN single‐cell suspensions were restimulated with 15 μg/ml HDM for 3 days to measure cytokine production in culture medium by ELISA. *N* = 6 mice per group.Data information: Data were analyzed with an unpaired Kruskal–Wallis test without multiple comparison correction and are shown as means ± SEM. *P < 0.05; **P < 0.01; ***P < 0.001; ****P < 0.0001 compared to β‐gal—PBS. ^$^ *P* < 0.01; ^$$^ *P* < 0.001 compared to α‐CD4—HDM. All exact *P*‐values are presented in Table EV1.

**Figure EV3:**
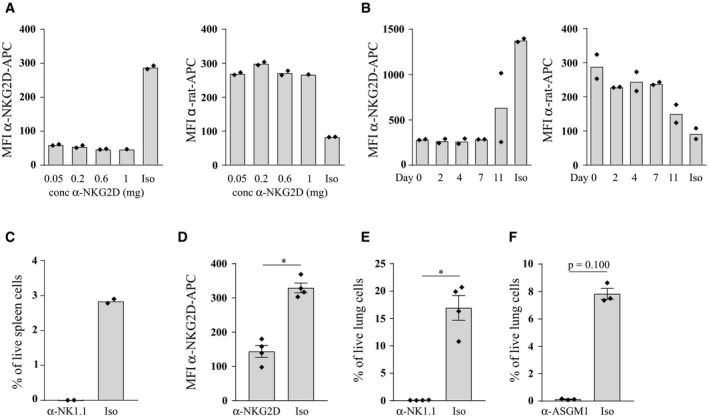
Efficient cell‐surface NKG2D blocking or NK cell depletion by intraperitoneal administration of anti‐NKG2D, anti‐NK1.1, or anti‐ASGM1 antibodies AC57Bl/6J WT mice received different concentrations of rat anti‐NKG2D blocking (CX5) or isotype (Iso) control antibodies intraperitoneally (i.p.) once, and were sacrificed 3 days later. Occupation of the NKG2D receptor by this blocking antibody was determined by staining of splenic NK cells with a fluorochrome‐conjugated antibody of the same clone (α‐NKG2D‐APC) (left panel), or by staining with an anti‐rat antibody (α‐rat‐APC) (right panel).BMice were i.p. administered 200 μg of anti‐NKG2D blocking antibody once and sacrificed at indicated time points for the assessment of NKG2D blocking on splenic NK cells by flow cytometry as explained in (A).CNK cell depletion in spleens 3 days after a single i.p. administration of anti‐NK1.1 antibody, determined by flow cytometry.D–FC57Bl/6J WT mice were sensitized intratracheally on day 0 with 1 μg HDM, or mock‐sensitized with PBS, followed after 1 week by five consecutive intranasal challenges with 10 μg HDM. From day −1, NKG2D‐blocking anti‐NKG2D (D), or NK cell‐depleting anti‐NK1.1 (E) or anti‐ASGM1 (F) antibodies, were i.p. administered every 3–4 days. 4 days after the last HDM challenge, NKG2D blocking on NK cells (D) or NK cell depletion (E, F) in lung tissue was confirmed by flow cytometry.Data information: *N* per group = 2 (A–C), 1 (concentration 1 mg in A), 4 (D, E), or 3 (F). Data (D‐F) were analyzed with an unpaired Mann–Whitney *U*‐test and shown as individual data points with the means (±SEM). *P = 0.0286.

**Figure EV4:**
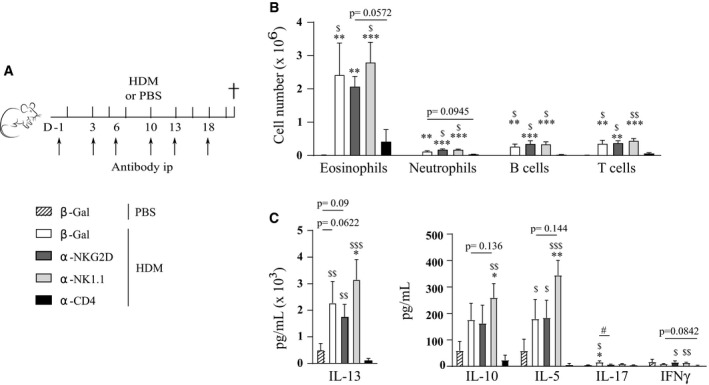
Allergic asthma, induced by chronic HDM exposure, is not influenced by antibody‐mediated depletion of NK cells or NKG2D blocking on NK cells C57Bl/6J WT mice were intranasally challenged with 25 μg HDM three times a week for 3 weeks, or mock‐challenged with PBS. During this protocol, they received i.p. injections of anti‐NKG2D blocking antibodies (CX5), cell‐depleting antibodies against NK1.1 (PK136), CD4^+^ cell‐depleting antibodies (GK1.5), or control anti‐β‐galactosidase antibodies on indicated time points.Infiltration of eosinophils, neutrophils, B cells, and T cells to BAL, 24 h after the last challenge, assessed by flow cytometry.MLN single‐cell suspensions were restimulated with 15 μg/ml HDM for 3 days, and cytokine production was measured by ELISA.Data information: *N* = 6 mice per group. Data were analyzed with an unpaired Kruskal–Wallis test without multiple comparison correction and are shown as means ± SEM. *P < 0.05; **P < 0.01; ***P < 0.001 compared to β‐Gal—PBS group. ^$^ *P* < 0.05; ^$$^ *P* < 0.01; ^$$$^ *P* < 0.001 compared to α‐CD4—HDM group. ^#^ *P* < 0.05. All exact *P*‐values are presented in Table EV1.

**Figure 7:**
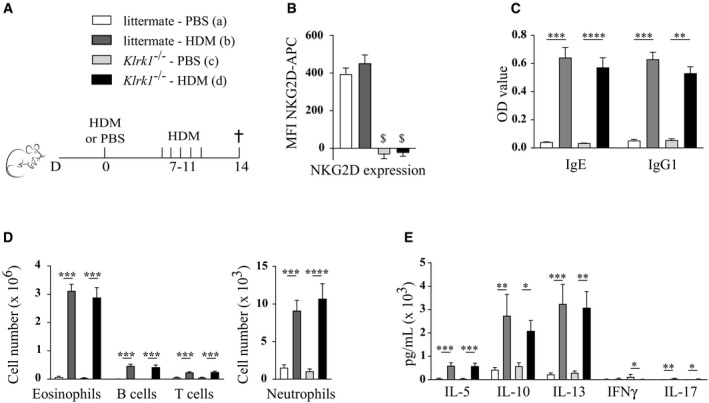
Absence of NKG2D surface expression does not influence HDM‐induced allergic asthma C57Bl/6 NKG2D knockout mice (Klrk1^−/−^) or littermate controls were sensitized intratracheally on day 0 with 1 μg HDM, or mock‐sensitized with PBS, followed after 1 week by five consecutive intranasal challenges with 10 μg HDM, and analysis 3 days after the last challenge.Mean fluorescence intensity (MFI) of NKG2D‐APC on live splenic NK cells (CD3^−^ NK1.1^+^ CD122^+^), assessed by flow cytometry.HDM‐specific immunoglobulin serum levels, detected by ELISA.Infiltration of eosinophils, B cells, T cells, and neutrophils to BAL, determined using flow cytometry.MLN single‐cell suspensions were restimulated with 15 μg/ml HDM for 3 days to measure cytokine production in culture medium by ELISA.Data information: All data are pooled from two independent experiments with total *n* = 7 (a), 15 (b), 8 (c) or 14 (d). Data were analyzed with an unpaired Kruskal–Wallis test without multiple comparison correction and are shown as means ± SEM. *P < 0.05; **P < 0.01; ***P < 0.001; ****P < 0.0001. ^$^ *P* < 0.001 compared to both littermate groups. All exact *P*‐values are presented in Table EV1.

**Figure EV5:**
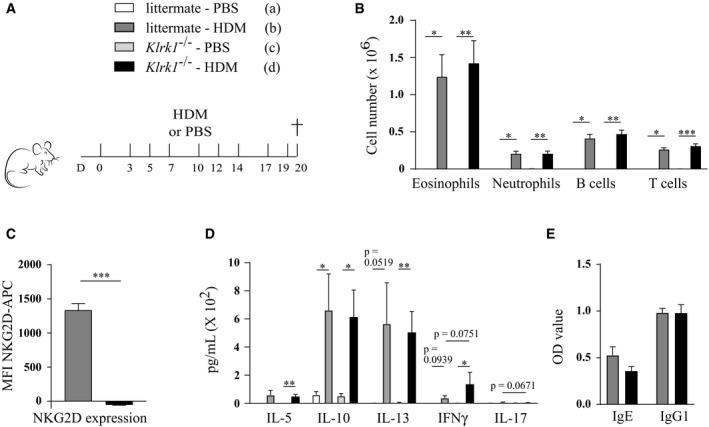
Absence of NKG2D surface expression does not significantly influence allergic asthma in response to chronic HDM exposure Mice genetically deficient for NKG2D (Klrk1^−/−^) or littermate controls were intranasally challenged with 25 μg HDM three times a week for 3 weeks and analyzed 24 h later.Infiltration of eosinophils, neutrophils, B cells, and T cells to BAL, determined using flow cytometry.Mean fluorescence intensity (MFI) of NKG2D‐APC on live splenic NK cells (CD3^−^ NK1.1^+^ CD122^+^), assessed by flow cytometry.MLN single‐cell suspensions were restimulated with 15 μg/ml HDM for 3 days to measure cytokine production in culture medium by ELISA.HDM‐specific immunoglobulin serum levels, detected by ELISA.Data information: In (B and D), data are pooled from two independent experiments with total *n* = 3 (a), 4 (c), or 15 (b, d). In (C and E), *n* = 8 per group and data are representative of two independent experiments. Data were analyzed with an unpaired Kruskal–Wallis test without multiple comparison correction and are shown as means ± SEM. *P < 0.05; **P < 0.01; ***P < 0.001. All exact *P*‐values are presented in Table EV1.

To facilitate a careful comparison with earlier studies, which utilized NK cell‐depleting antibodies, we treated C57Bl/6 wild‐type mice with anti‐NK1.1 or anti‐ASGM1 depleting antibodies, anti‐NKG2D blocking antibodies, anti‐β‐galactosidase antibodies as a control, or anti‐CD4 antibodies to deplete CD4^+^ T cells as an additional control (Fig 6A). Anti‐NKG2D blocking antibody efficiently blocked NKG2D surface expression on splenic NK cells for at least 7 days (Fig EV3A and B). As depleted NK cells start to reconstitute after 4 days (Fig EV1A), all antibodies were administered every 3–4 days. NKG2D blocking or complete NK cell absence was again confirmed in the lungs at the time of sacrifice (Fig EV3C–F). Whereas CD4^+^ T cell depletion completely aborted influx of eosinophils in the BAL, administration of the other antibodies did not inhibit asthma hallmarks (Fig 6B–D). There was a trend for higher inflammatory cell influx in anti‐ASGM1‐treated animals, but this failed to reach statistical significance. Moreover, administration of anti‐NK1.1 or anti‐NKG2D also did not alter cardinal features of allergic asthma induced by the chronic HDM‐based asthma model (Fig EV4A–C). The finding that administration of anti‐NKG2D does not inhibit HDM‐mediated allergic asthma contrasts with earlier research that used mice genetically deficient for NKG2D (*Klrk1* ^−/−^) (Farhadi *et al*, 2014). Accurate comparison of these results might be hampered by some undesirable side effects of antibody injection, such as nonspecific binding to Fc receptors, or surface receptor cross‐linking followed by cell activation (Arase *et al*, 1997). To eliminate confusion from these side effects, *Klrk1* ^−/−^ mice and littermate controls were subjected to HDM‐induced asthma (Fig 7A and B). This resulted in substantial and comparable HDM‐specific IgE and IgG1 serum levels (Fig 7C), infiltration of eosinophils and other immune cells in the BAL (Fig 7D), and Th2 cytokine production by HDM‐restimulated MLN cells (Fig 7E), in both NKG2D‐deficient and littermate mice. Finally, genetic absence of NKG2D had no impact on BAL eosinophilia, Th2 cytokine production, or IgE and IgG1 serum levels in response to chronic HDM exposure (Fig EV5A–E).

## Discussion

In this study, we comprehensively addressed the long‐held hypothesis that NK cells play an important role in asthma. Using the most advanced method to genetically deplete NKp46^+^ NK cells, we found no support for this hypothesis. Therefore, the claim of a true null hypothesis (NK cells do not play a critical role in asthma) was further substantiated. NK cells were depleted during asthma development by the administration of conventional NK cell‐depleting antibodies. NK cells were also temporarily depleted during the separate phases of asthma development (sensitization and challenge), eliminating the possibility that the role of NK cells during these phases might be opposite. To address any potential confounding bias due to the type of animal model used, NK cells were depleted in an OVA/alum‐based model and a more chronic HDM‐driven model. Finally, as cellular depletion could not be the best option to reveal a role for NK cells, we interfered with NK cell function rather than numbers by blocking the prototypic NK cell‐activating NKG2D receptor using antibodies or genetic NKG2D deficiency. As none of these intervention strategies affected asthma severity, we accept the null hypothesis that NK cells are dispensable for allergic asthma development in mice.

NKp46 is not entirely specific for NK cells and is also expressed on Eomes^+^ ILC1s in salivary glands, Eomes^−^ ILC1s in liver, skin, and peritoneum, and a subset of IL‐22 producing ROR‐γt^+^ ILC3s in the gut (Cella *et al*, 2009; Luci *et al*, 2009; Sanos *et al*, 2009; Cortez & Colonna, 2016). These populations are very rare in the lungs of naive mice, but the ILC1 population could potentially expand in inflamed tissues, as was shown for CD127^+^ ILC1‐like cells in the spleens of chronically infected mice (Gasteiger *et al*, 2013). Additionally, NKp46 expression has been detected on a subset of non‐CD1d restricted NK1.1^+^ NKT cells (Walzer *et al*, 2007; Yu *et al*, 2011) and a subset of γδT cells (Stewart *et al*, 2007). Our data suggest that all these cell (sub)populations are also dispensable for HDM‐mediated asthma development.

Our findings are in marked contrast to earlier studies that have relied on targeting NK1.1, ASGM1, or NKG2D (Korsgren *et al*, 1999; Ple *et al*, 2010; Ghadially *et al*, 2013; Farhadi *et al*, 2014), none of which are unique for NK cells. NK1.1 is expressed on both NK and NKT cells, the latter possibly being able to modulate airway disease (Akbari *et al*, 2003; Berzins & Ritchie, 2014), and on some virus‐specific CD4^+^ and CD8^+^ T cells (Slifka *et al*, 2000). ASGM1 has been detected on the majority of, but not all, murine NK cells, subsets of CD8^+^ T cells, basophils, and in some instances even on eosinophils or activated CD4^+^ T cells (Trambley *et al*, 1999; Slifka *et al*, 2000; Kataoka *et al*, 2004; Nishikado *et al*, 2011). We demonstrated NKG2D expression on NK cells, but also on activated CD44^+^ CD4^+^ T cells, albeit to a lesser extent. Moreover, NKG2D expression was detected on NKT cells, γδT cells, and CD8^+^ T cells (Bauer, 1999; Jamieson *et al*, 2002). Nevertheless, in the two HDM models investigated here, depletion of NK1.1‐ or ASGM1‐expressing cells did not blunt allergic inflammation, nor did the antibody‐mediated blocking or genetic removal of the activating NKG2D receptor. Therefore, this study excludes differences in experimental techniques as an explanation for conflicting results and affirms our conclusions across several models of NK cell depletion or targeting.

Recently, interactions between NK cells and ILC2s have been demonstrated in HDM‐induced allergic asthma, in which NK cells play an anti‐inflammatory role (Ferrini *et al*, 2016; Simons *et al*, 2017). The absence of the eicosanoid CB2 or PGI_2_ receptor resulted in increased NK cell numbers in BAL, indirectly affecting ILC2s numbers and activity, and impediment of asthma development. By means of transfer studies, this was validated to be NK cell dependent. Notably, this anti‐inflammatory role of NK cells was only observed in conditions where NK cells were strongly increased in number and possibly differentially activated. Indeed, WT mice, harboring physiological amounts of pulmonary NK cells, were perfectly able to develop inflammation upon HDM instillation, and anti‐NK1.1 antibody treatment in these mice caused only a modest increase in total BAL cell numbers without influencing other investigated asthma hallmarks (Ferrini *et al*, 2016; Simons *et al*, 2017). Interestingly, the rare human NK cell deficiencies described in literature have not yet been associated with hypersensitivity reactions (Voss & Bryceson, 2017). Although the authors justly state that modulating NK cell function provides a promising therapeutic strategy, we here show that NK cells, when present in the physiological amounts and activation state imposed by HDM exposure, have negligible anti‐inflammatory capacities.

Interestingly, none of the studies that demonstrated a pro‐inflammatory function of NK cells mentioned the pathogen status of their animal facility. Our mouse studies were performed in specific pathogen free (SPF)‐housed animals. Differences in housing conditions could potentially have an impact on gut microbiome diversity, which, in humans, has been shown to correlate with increased risk of atopic eczema or allergic asthma (Bisgaard *et al*, 2011; Abrahamsson *et al*, 2012). The risk of allergen sensitization might also directly be influenced by housing conditions; we previously showed that exposure to minute amounts of LPS or farm dust drastically blunted the allergic airway response, due to desensitization of the airway epithelium (Schuijs *et al*, 2015). Moreover, mice fed a high‐fiber diet had a changed composition of gut and lung microbiome and were protected from allergic asthma development (Trompette *et al*, 2014). Therefore, the threshold for allergic sensitization could be increased in animal facilities in which animals have a different microbiome composition or are fed a different animal chow, or in which some pathogens are allowed to persist. This may then possibly necessitate additional innate signals, such as those delivered by NK cells, to develop a full‐blown allergic response.

In summary, by using the newest genetic tools, we demonstrate that NK cells play a minor role in the establishment of HDM‐induced allergic asthma. In the future, we have to more comprehensively assess the impact of environmental conditions and microbiome on the function of NK cells in asthma. Only then, we will be able to model and grasp the full therapeutic potential of these cells in human asthma.

## Materials and Methods

### Mice

*Ncr1* ^iCre/iCre^ mice were previously described (Narni‐Mancinelli *et al*, 2011). *ROSA* ^DTA/DTA^, *ROSA* ^DTR/DTR^, and *Klrk1* ^−/−^ (Guerra *et al*, 2008) mice were obtained from The Jackson Laboratory. C57Bl/6J wild‐type mice were obtained from Janvier. All mice were on a C57Bl/6 genetic background and 6–10 weeks old. As differences in male and female mice were not observed, both sexes were used and the mice were first age‐ and sex‐matched, then randomly assigned per group and per experiment, without specific randomization procedure. Mice were housed and bred at the specific pathogen free (SPF) facility of VIB‐University of Ghent, in individually ventilated cages with a controlled day–night cycle and given food and water *ad libitum*. Experiments conform to the Belgian laws and regulations concerning the use of animals for research and were approved by the Animal Ethical Committees of the University of Ghent and the Center of Inflammation Research.

### Models of allergic asthma

NK cell kinetics following HDM exposure were investigated by i.t. instilling mice with 100 μg HDM. Acute HDM‐induced allergic inflammation was established by i.t. sensitization with 1 μg HDM extract (Greer Laboratories), or with PBS as a control (day 0), followed by five consecutive i.n. challenges with 10 μg HDM (days 7–11). Asthma features were analyzed 3 or 4 days later. In the chronic HDM‐induced asthma model adapted from (Johnson *et al*, 2004; Gregory *et al*, 2009), mice were instilled i.n. with 25 μg HDM, or PBS as a control, three times a week for 3 weeks. Asthma features were determined 24 h after the last challenge. OVA‐mediated asthma was induced by an i.p. injection of 10 μg purified OVA (Worthington) absorbed on 1 mg alum (day 0), a boost i.p. injection of 10 μg purified OVA (day 7), and subsequent i.n. challenges on days 17–19 with 20 μg OVA. Asthma features were determined 24 h after the last challenge. All i.t. and i.n. treatments were given in 80 and 40 μl PBS, respectively, and under light isoflurane anesthesia.

### *In vivo* ablation of cells or blocking of NKG2D

To deplete NKp46^+^ cells in *ROSA* ^DTR/+^ *Ncr1* ^iCre/+^ mice, 200 ng DT (Sigma) was injected intravenously at indicated time points. For antibody‐mediated depletion or blocking studies, mice were i.p. administered 200 μg of antibodies, diluted in PBS, every 3–4 days, starting at day −1. Anti‐NKG2D (CX5), anti‐CD4 (GK1.5), anti‐NK1.1 (PK136), and control anti‐β‐galactosidase (GL113) antibodies were produced by Bioceros. Anti‐ASGM1 was purchased from Wako and 50 μl of reconstituted (in 1 ml dH_2_O) antibodies were administered, diluted in PBS.

### Effector cytokine production

Dissected MLNs were pressed through a 100‐μM cell sieve. The acquired single‐cell suspensions were seeded (2 × 10^6^ cells/ml) in 96‐well plates in RPMI‐1640 medium supplemented with 5% fetal calf serum (Bodinco), 0.1% β‐mercaptoethanol, glutamax (Gibco) and gentamycin (Gibco), and restimulated with 15 μg/ml HDM for 3 days. Snap‐frozen total lungs were homogenized in a tissue Lyser II device (Qiagen) for 4 min at 20 Hz, in 20% glycerol in dH_2_O with 40 mM Tris–HCl, 275 mM NaCl, and an EasyPack complete ULTRAtablet mini (Roche). 2% Igepal CA‐630 (US biologicals) was added, and homogenates were rotated for 30 min and then centrifuged. MLN culture and homogenized lung tissue supernatants were analyzed for cytokine levels by ELISA (Ready‐set‐go kits from eBioscience), and for total protein concentration with NanoOrange technology (Thermo Fisher, Invitrogen).

### Immunoglobulin production

Mice were bled under terminal anesthesia, and serum was collected by centrifugal phase separation to determine IgE and IgG1 levels by ELISA (BD Biosciences). For HDM‐specific IgG1, ELISA plates were coated with 100 μg/ml HDM (Greer Laboratories); For HDM‐specific IgE, the supplemented detection antibody was interchanged for biotin‐labeled HDM (100 μg/ml), diluted in PBS + 10% FCS.

### Flow cytometry

Bronchoalveolar lumen fluid was obtained by flushing the lungs with EDTA‐containing PBS (0.5 mM) via a cannula inserted in the trachea. Spleens and MLNs were dissected and pressed through a 100‐μM cell sieve. Bones were crushed with mortar and pestle in RPMI‐1640 medium and filtered through a 70‐μM cell sieve. Whole lungs were isolated in RPMI‐1640 medium supplemented with DNAse I recombinant Grade I (10 U/ml) and Liberase TM (20 μg/ml), both purchased from Roche. Lung tissue was dissociated using the GentleMACS (Miltenyi Biotec) lung programs 1 and 2, with gentle shaking at 37°C for 30 min in between both steps. The reaction was stopped by adding excess PBS, and the obtained single‐cell suspensions were filtered through a 100‐μm sieve. Cell suspensions were treated with osmotic lysis buffer, stained with antibody cocktails in PBS for 30 min at 4°C, and subsequently washed in PBS supplemented with 2 mM EDTA, 0.5% BSA, and 0.01% sodium azide. Unspecific antibody binding was prevented by adding 2.4G2 (antibody to the Fcγ receptor II/III) during the staining. Dead cells were excluded by adding fixable viability dye conjugated to eFluor506 (eBioscience). A fixed amount of counting beads (123count ebeads, Thermo Fisher Scientific) was added to determine absolute cell numbers. Antibodies used for flow cytometry are summarized in Table EV2. Samples were acquired on a LSRFortessa (4 laser, BD Biosciences) and analyzed using Flowjo Software (Tree Star, Inc). In BAL, eosinophils were gated as CD11c‐ CD3/19‐ Ly6G‐ CD11b^hi^ SiglecF^hi^ SSC‐A^hi^, neutrophils as CD11c‐ CD3/19‐ Ly6G^hi^ CD11b^hi^, B cells as CD11c‐ CD3/19^hi^ MHC‐II^hi^ and T cells as CD11c‐ CD3/19^hi^ MHC‐II^−^.

### Mucus production

Lungs were inflated with 1 ml PBS/OCT (1:1) solution (Tissue‐Tek), snap‐frozen in liquid nitrogen, and cryosectioned (7 μm) using the HM560 microtome (Thermo Scientific) for PAS staining. Pictures were obtained with AnalySIS getIT (Olympus Soft Imaging Solutions).

### BHR determination

Mice were anesthetized with urethane, paralyzed with D‐tubocurarine, tracheotomized, and intubated with a 28‐G catheter, followed by mechanical ventilation in a Flexivant apparatus (SCIREQ). Respiratory frequency was set at 150 breaths/min with a tidal volume of 10 ml/kg, and a positive‐end expiratory pressure of 3 cm H_2_O was applied. Increasing concentrations of methacholine were nebulized (0–400 mg/ml); baseline resistance was restored in between the doses. A standardized inhalation maneuver was given every 10 s for 2 min per dose, and dynamic resistance and compliance were recorded.

### Statistics

Sample sizes (*n*) represent the number of independent biological replicates and were chosen according to previous experience with the used experimental asthma models. Investigators were not blinded during the experiments and/or analyses. The differences between two, or more, groups were calculated with the Mann–Whitney *U*‐test for unpaired data, or the Kruskal–Wallis test for unpaired data, respectively, without multiple comparison correction (GraphPad Prism version 7.0; GraphPad, San Diego, CA). These non‐parametric statistical tests were chosen because data were not normally distributed, and/or because normal distribution of the data sets could not be assumed due to too small sample sizes and/or unequal variance between experimental groups. Data are shown as means ± SEM, or as individual data points, where *n* < 5. *N*‐values and statistical tests used are mentioned in the figure legends; all relevant *P*‐values < 0.15 are depicted in the figures. All exact *P*‐values are represented in Table EV1.

## Author contributions

Conceptualization: MJvH, EH, EV, and BNL; methodology: MJvH, EH, EV; investigation: EH, MJvH, KD, SDP, and JvM; writing—original draft: EH; writing—review and editing: MJvH, EV, BNL, and HH; funding acquisition: EH, MJvH, BNL, and HH; resources: BNL, HH, EV, and LB; supervision: BNL, HH, and EV.

## Conflict of interest

Disclosure of potential conflict of interest: Prof. Dr. Eric Vivier is co‐founder, scientific advisor, and shareholder of Innate‐Pharma. The other authors declare that they have no conflict of interest.

**The paper explained**

**Problem**

Allergic asthma is a major health problem that currently affects 300 million people worldwide. A thorough understanding of the underlying pathogenesis is key to the development of curing therapies. Asthma is mainly driven by T helper 2 lymphocytes, but several cells of the innate immune system also control key aspects of the disease. The role of conventional natural killer (NK) cells, however, remains elusive. Although NK cells have been proposed as a suitable target for asthma therapy, murine studies on their function in asthma are conflicting, in part because different, not entirely specific, tools have been employed to deplete NK cells or target their function.

**Results**

In mice that were given inhaled house dust mite, a clinically relevant allergen, NK cells migrated to the lungs and lung‐draining lymph nodes. However, *Ncr1*‐DTA mice, which are genetically engineered to lack all NK cells in the most specific way to date, still robustly developed all asthma symptoms upon acute or chronic house dust mite exposure. Temporary depletion of NK cells during separate phases of asthma development in *Ncr1*‐DTR mice also had no impact on the inflammatory phenotype. The results were additionally confirmed by injecting conventional NK cell‐depleting antibodies. Finally, antibody‐mediated blocking of the NK cell‐activating NKG2D receptor, or genetic NKG2D deficiency, did not alter asthma severity.

**Impact**

This study challenges earlier findings and demonstrates that NK cells are not necessary for house dust mite‐dependent asthma development. It thus suggests that NK cells may not be the most robust and all‐encompassing therapeutic target to tackle allergic asthma. Alternatively, it suggests that we may need a more comprehensive assessment of the influence of environmental and microbial conditions on the precise role of NK cells in asthma.

## Supporting information



Expanded View Figures PDF

Table EV1

Table EV2

Review Process File
