# Supplementary material for: Role of NKp46+ natural killer cells in house dust mite‐driven asthma
Source: EMBO Mol Med. 2018 Feb 15;10(4):e8657. doi: 10.15252/emmm.201708657 (PMC5887908; doi:10.15252/emmm.201708657)
Supplement: Supplementary file 1 — Expanded View Figures PDF [file EMMM-10-e8657-s001.pdf]

Expanded View Figures

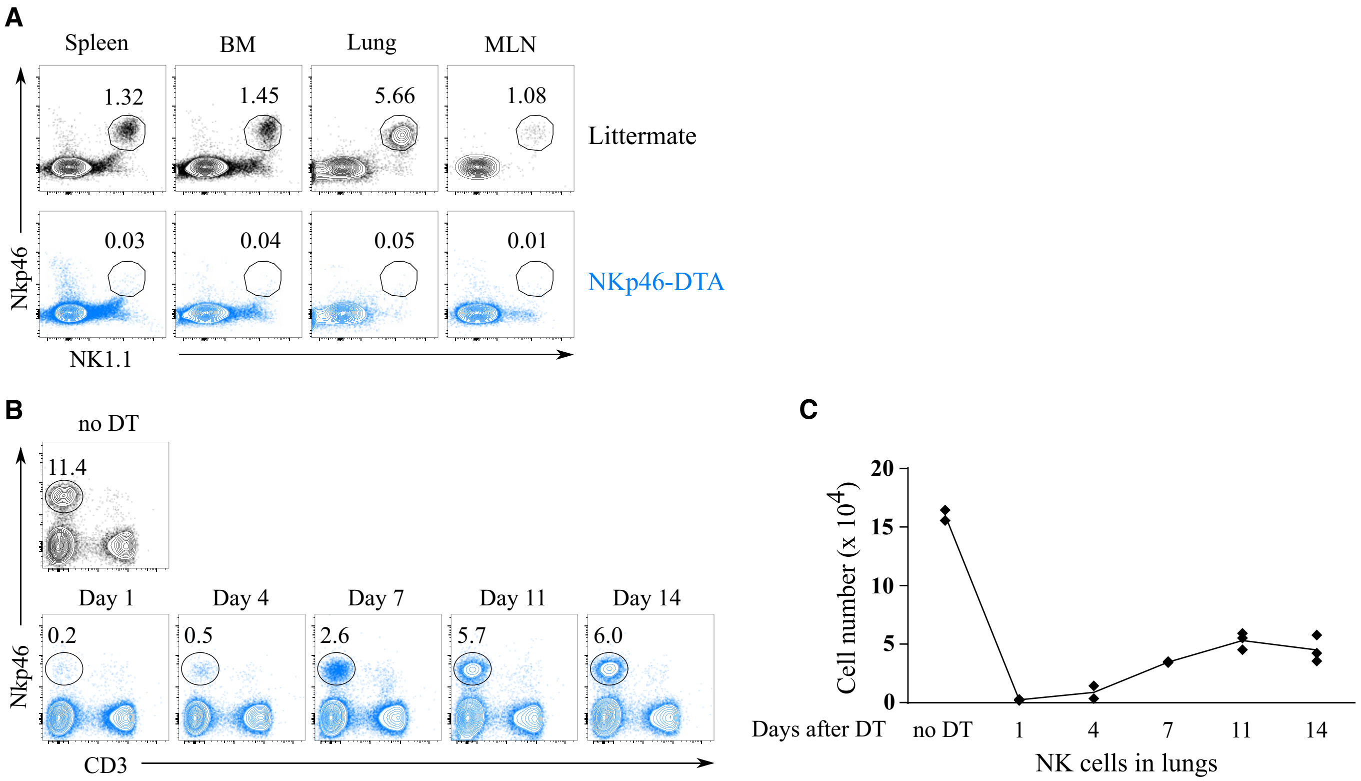

Figure EV1. NK cells are efficiently depleted in *Ncr1*$^{iCre/+}$ *ROSA*$^{DTA/+}$ mice and can efficiently be depleted by DT injection in *Ncr1*$^{iCre/+}$ *ROSA*$^{DTR/+}$ mice.

A Flow cytometry analysis of NK cells in spleen, bone marrow (BM), lung, and mediastinal lymph nodes (MLN) isolated from naïve NKp46-DTA (*Ncr1*$^{iCre/+}$ *ROSA*$^{DTA/+}$) mice or littermate controls (*Ncr1*$^{iCre/+}$ *ROSA*$^{+/+}$). First panel was pre-gated on live CD45^{+}CD3^{-} single cells.

B, C NKp46-DTR (*Ncr1*$^{iCre/+}$ *ROSA*$^{DTR/+}$) mice were treated with 200 ng DT intravenously and sacrificed at indicated time points. (B) Representative flow cytometry plots and (C) quantification of NK cell numbers (live, CD3^{-} NKp46^{+}) in lung tissue cell suspensions. Gated on live single cells. *N* = two to three mice per time point and means are connected.

 © 2018 The Authors

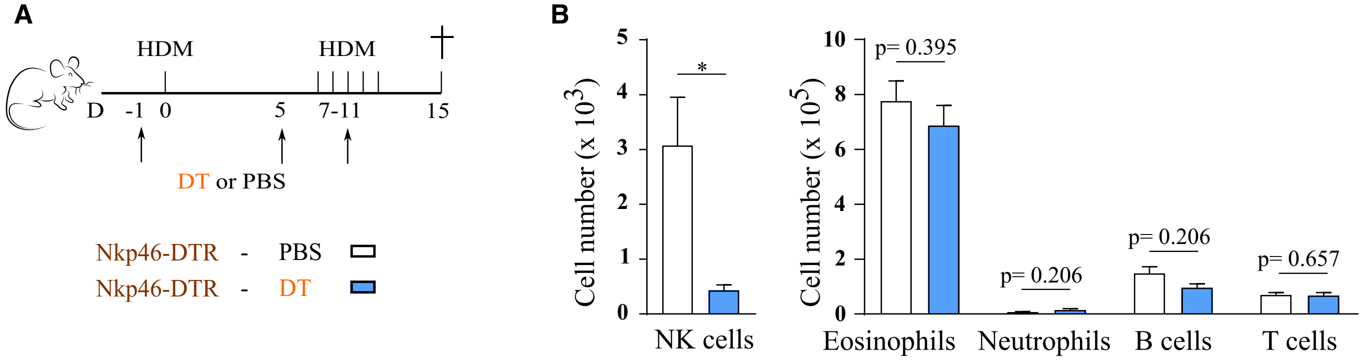

Figure EV2. Ablation of NKp46$^+$ cells during the entire HDM-induced allergic asthma protocol does not influence immune cell infiltration to the BAL.

A NKp46-DTR mice (*Ncr1*$^{iCre/+}$ *ROSA*$^{DTR/+}$) were sensitized intratracheally on day 0 with 1 μg HDM, and 1 week later, they were intranasally challenged on five consecutive days with 10 μg HDM. DT was injected at indicated time points to deplete NKp46$^+$ cells.

B Infiltration of NK cells, eosinophils, neutrophils, B cells, and T cells to BAL, determined by flow cytometry. Data are pooled from two independently performed experiments with total n = 11 (PBS group) or 8 (DT group). Data were analyzed with an unpaired Mann-Whitney U-test and are shown as means ± SEM. *P = 0.0157.

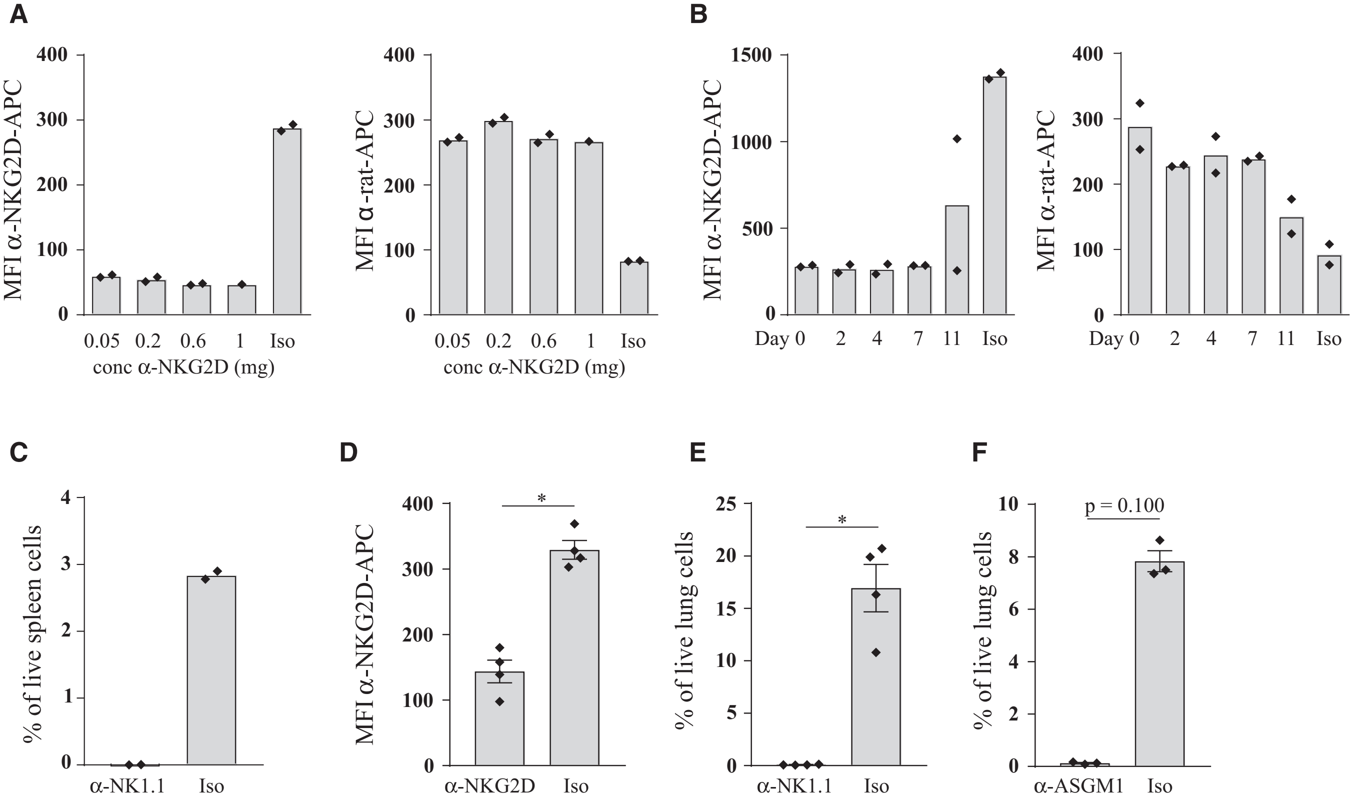

Figure EV3. Efficient cell-surface NKG2D blocking or NK cell depletion by intraperitoneal administration of anti-NKG2D, anti-NK1.1, or anti-ASGM1 antibodies.

A C57Bl/6J WT mice received different concentrations of rat anti-NKG2D blocking (CX5) or isotype (Iso) control antibodies intraperitoneally (i.p.) once, and were sacrificed 3 days later. Occupation of the NKG2D receptor by this blocking antibody was determined by staining of splenic NK cells with a fluorochrome-conjugated antibody of the same clone (α-NKG2D-APC) (left panel), or by staining with an anti-rat antibody (α-rat-APC) (right panel).

B Mice were i.p. administered 200 μg of anti-NKG2D blocking antibody once and sacrificed at indicated time points for the assessment of NKG2D blocking on splenic NK cells by flow cytometry as explained in (A).

C NK cell depletion in spleens 3 days after a single i.p. administration of anti-NK1.1 antibody, determined by flow cytometry.

D–F C57Bl/6J WT mice were sensitized intratracheally on day 0 with 1 μg HDM, or mock-sensitized with PBS, followed after 1 week by five consecutive intranasal challenges with 10 μg HDM. From day −1, NKG2D-blocking anti-NKG2D (D), or NK cell-depleting anti-NK1.1 (E) or anti-ASGM1 (F) antibodies, were i.p. administered every 3–4 days. 4 days after the last HDM challenge, NKG2D blocking on NK cells (D) or NK cell depletion (E, F) in lung tissue was confirmed by flow cytometry.

Data information: *N* per group = 2 (A–C), 1 (concentration 1 mg in A), 4 (D, E), or 3 (F). Data (D-F) were analyzed with an unpaired Mann–Whitney *U*-test and shown as individual data points with the means (±SEM). $^*P = 0.0286$.

 © 2018 The Authors

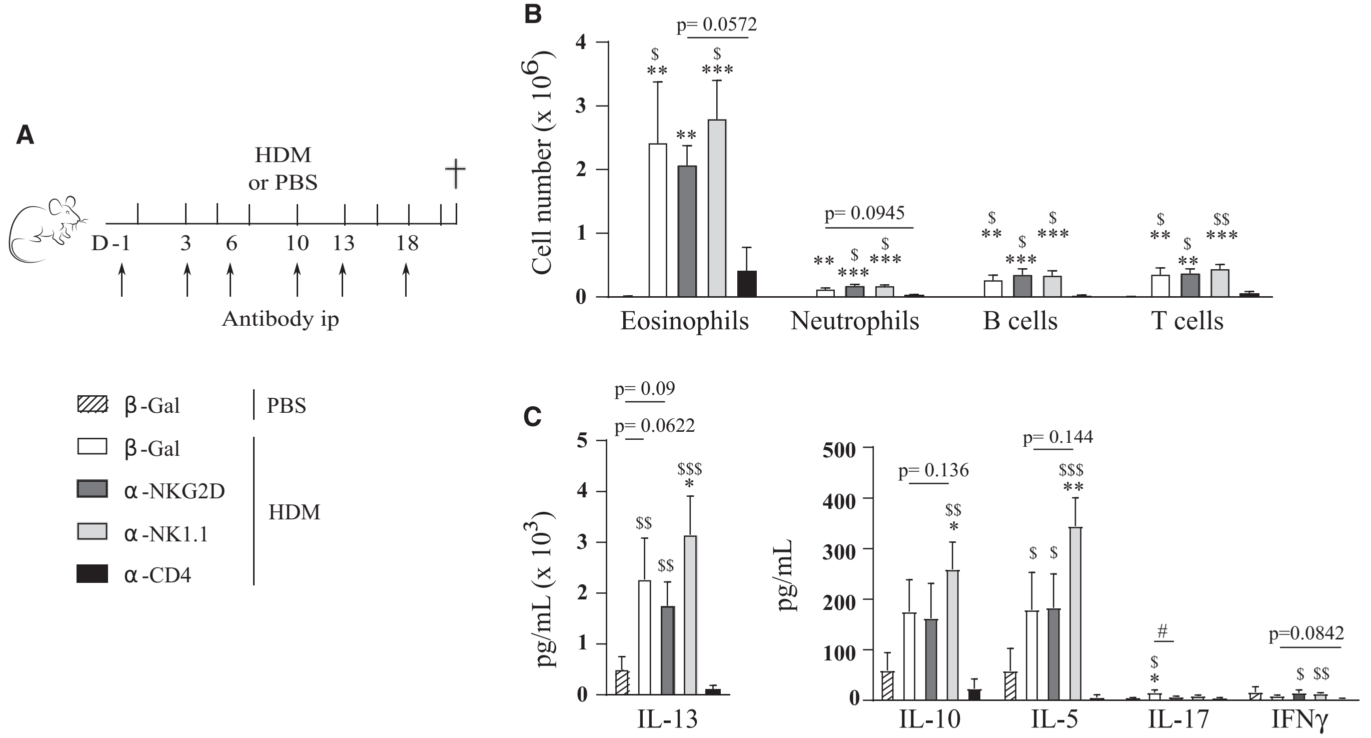

Figure EV4. Allergic asthma, induced by chronic HDM exposure, is not influenced by antibody-mediated depletion of NK cells or NKG2D blocking on NK cells.

A C57Bl/6J WT mice were intranasally challenged with 25 µg HDM three times a week for 3 weeks, or mock-challenged with PBS. During this protocol, they received i.p. injections of anti-NKG2D blocking antibodies (CX5), cell-depleting antibodies against NK1.1 (PK136), $CD4^+$ cell-depleting antibodies (GK1.5), or control anti-β-galactosidase antibodies on indicated time points.

B Infiltration of eosinophils, neutrophils, B cells, and T cells to BAL, 24 h after the last challenge, assessed by flow cytometry.

C MLN single-cell suspensions were restimulated with 15 µg/ml HDM for 3 days, and cytokine production was measured by ELISA.

Data information: $N = 6$ mice per group. Data were analyzed with an unpaired Kruskal–Wallis test without multiple comparison correction and are shown as means ± SEM. $^*P < 0.05$; $^{**}P < 0.01$; $^{***}P < 0.001$ compared to β-Gal—PBS group. $^{\$}P < 0.05$; $^{\$\$}P < 0.01$; $^{\$\$\$}P < 0.001$ compared to α-CD4—HDM group. $^{\#}P < 0.05$. All exact *P*-values are presented in Table EV1.

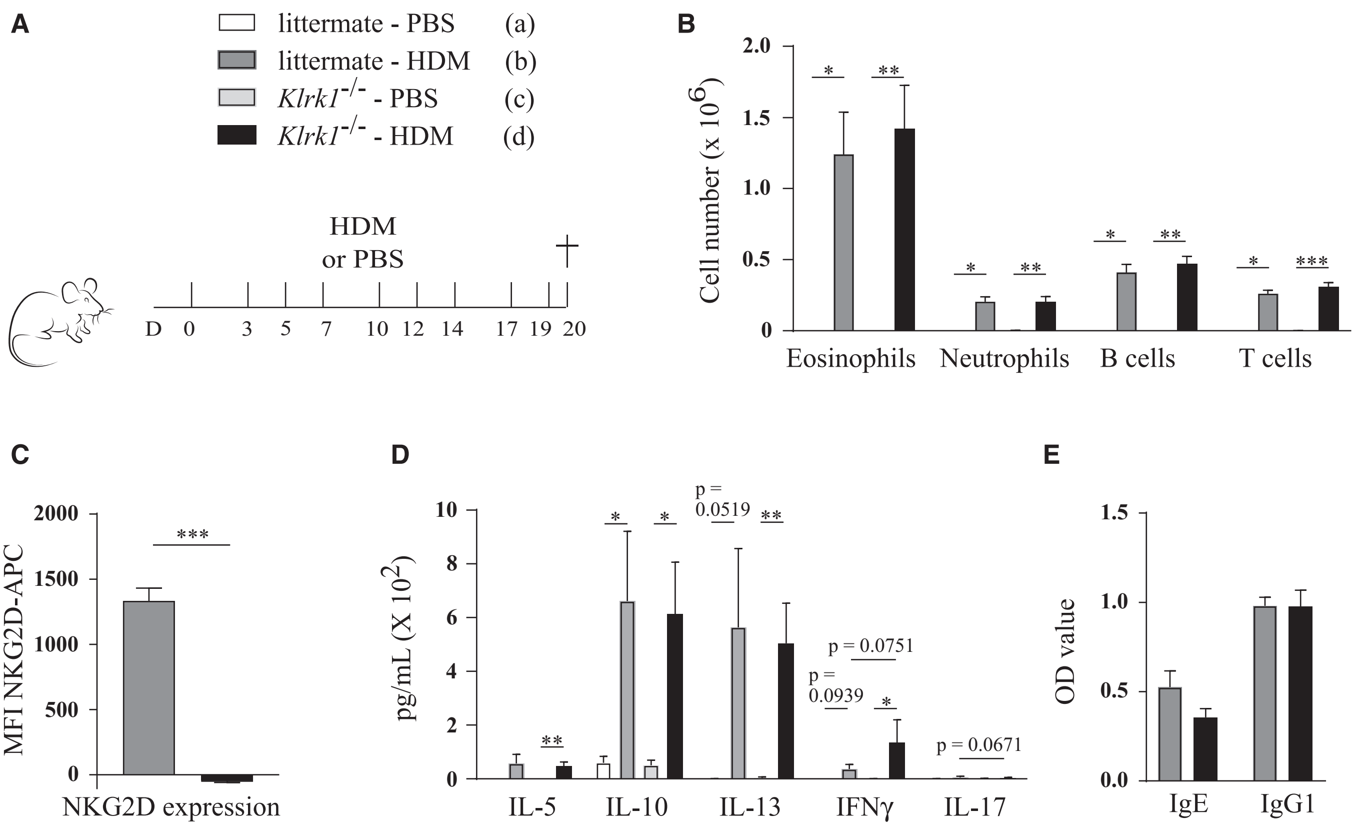

Figure EV5. Absence of NKG2D surface expression does not significantly influence allergic asthma in response to chronic HDM exposure.

A Mice genetically deficient for NKG2D (Klrk1$^{-/-}$) or littermate controls were intranasally challenged with 25 μg HDM three times a week for 3 weeks and analyzed 24 h later.

B Infiltration of eosinophils, neutrophils, B cells, and T cells to BAL, determined using flow cytometry.

C Mean fluorescence intensity (MFI) of NKG2D-APC on live splenic NK cells (CD3^{-} NK1.1^{+} CD122^{+}), assessed by flow cytometry.

D MLN single-cell suspensions were restimulated with 15 μg/ml HDM for 3 days to measure cytokine production in culture medium by ELISA.

E HDM-specific immunoglobulin serum levels, detected by ELISA.

Data information: In (B and D), data are pooled from two independent experiments with total n = 3 (a), 4 (c), or 15 (b, d). In (C and E), n = 8 per group and data are representative of two independent experiments. Data were analyzed with an unpaired Kruskal–Wallis test without multiple comparison correction and are shown as means ± SEM. *$P < 0.05$; **$P < 0.01$; ***$P < 0.001$. All exact P-values are presented in Table EV1.

 © 2018 The Authors
