# Supplementary material for: Role of NKp46+ natural killer cells in house dust mite‐driven asthma
Source: EMBO Mol Med. 2018 Feb 15;10(4):e8657. doi: 10.15252/emmm.201708657 (PMC5887908; doi:10.15252/emmm.201708657)
Supplement: Supplementary file 3 — Table EV2 [file EMMM-10-e8657-s003.doc]

| **Marker** | **Fluorochrome** | **Clone** | **Company** | **Dilution** |
| --- | --- | --- | --- | --- |
| asialo-GM1 | AF647 | Polyclonal | Biolegend | 1/200 |
| CD11b | V450 | M1/70 | BD Biosciences | 1/800 |
| CD11c | PE-Cy7 | N418 | eBioscience | 1/500 |
| CD122 | PerCP-eFluor710 | TM-beta1 | eBioscience | 1/300 |
| CD19 | PE-Cy5 | 1D3 | eBioscience | 1/400 |
| CD3e | PE-Cy5 | 145-2C11 | Tonbo Biosciences | 1/200 |
| CD3 | AF700 | 17A2 | eBioscience | 1/150 |
| CD3 | PerCP-eFluor710 | 17A2 | eBioscience | 1/200 |
| CD4 | APC-Cy7 | GK1,5 | BD Biosciences | 1/200 |
| CD4 | AF700 | RM4-5 | eBioscience | 1/600 |
| CD44 | FITC | 5035-41.1D | Biorad | 1/50 |
| CD45 | APC-eFluor780 | 30-F11 | eBioscience | 1/200 |
| CD49b | eFluor450 | DX5 | eBioscience | 1/150 |
| CD69 | PerCP-Cy5.5 | H1,2F3 | BD Biosciences | 1/100 |
| Ly-6G | AF700 | 1A8 | BD Biosciences | 1/500 |
| MHCII | APC-eFlour780 | M5/114.15.2 | eBioscience | 1/800 |
| NK1.1 | PE | PK136 | BD Biosciences | 1/200 |
| NK1.1 | BV605 | PK136 | Biolegend | 1/300 |
| NKG2D | APC | CX5 | eBioscience | 1/100 |
| NKG2D | biotin | C7 | Biolegend | 1/50 |
| NKp46 | eFluor450 | 29A1.4 | eBioscience | 1/30 |
| Rat IgG | AF647 |  | Invitrogen | 1/500 |
| SiglecF | PE | E50-2440 | BD Biosciences | 1/1000 |
| Streptavidin | AF647 |  | Invitrogen | 1/3000 |
| TCRβ | APC-Cy7 | H57-597 | Biolegend | 1/200 |

Table E1: List of antibodies used for flow cytometry
